# Supplementary material for: A multimodal deep learning architecture for smoking detection with a small data approach
Source: Front Artif Intell. 2024 Feb 28;7:1326050. doi: 10.3389/frai.2024.1326050 (PMC10936563; doi:10.3389/frai.2024.1326050)
Supplement: Supplementary file 1 [file Data_Sheet_1.PDF]

Supplementary Material

1 DETAILED QUANTITATIVE RESULTS

Table S1. Validation accuracies for each pre-trained image processing network.

Model	Binary accuracy of validation
EfficientNet B0	0.9245
EfficientNet B1	0.9193
EfficientNet B2	0.8958
EfficientNet B3	0.9271
EfficientNet B4	0.9089
**EfficientNet B5**	**0.9375**
EfficientNet V2 B0	0.8906
EfficientNet V2 B1	0.8958
EfficientNet V2 B2	0.9167
EfficientNet V2 B3	0.9167
EfficientNet V2 M	0.9323
EfficientNet V2 S	0.9323
InceptionResNet V2	0.5182
Inception V3	0.6615
ResNet101 V2	0.5964
ResNet101	0.8490
ResNet152 V2	0.8698
ResNet152	0.8594
ResNet50 V2	0.6302
ResNet50	0.8490
vgg16	0.8385
vgg19	0.8672
Xception	0.6693

Table S2. Accuracy of multimodal filtering (MF), EfficientNetB5 (EB5), and ensembled multimodal filtering and EfficientNetB5 (MF-EB5) on the test videos.

Video	Full length in seconds	Real length of the smoking scene in seconds	Accuracy of MF	Accuracy of EB5	Accuracy of MF-EB5
Malboro 1	75	21	0.8919	0.6892	0.9054
Malboro 2	50	5	0.5200	0.4600	0.6600
Malboro 3	87	12	0.6781	0.6437	0.7931
Philip Moris 1	35	15	0.4286	0.5433	0.5710
Philip Moris 2	75	13	0.5730	0.6933	0.7600

Table S3. Results of a fine-tuned XLM RoBERTa for the named entity recognition task on the test data.

Loss	0.0736
Precision	0.9115
Recall	0.9035
F1 score	0.9075
Accuracy	0.9825

Table S4. The results of the language models on the validation data set.

Model	Loss	Precision	Recall	F1 score	Accuracy
XLM RoBERTa large	0.176	0.9580	0.9650	0.9615	0.9840
XLM RoBERTa base	0.140	0.9511	0.9669	0.9590	0.9829
huBERT	0.143	0.9326	0.9499	0.9412	0.9787
DistilBERT	0.101	0.9348	0.9597	0.9467	0.9784
BERT base multil. cased	0.096	0.9450	0.9666	0.9557	0.9821
BERT base multil. uncased	0.186	0.9170	0.9442	0.9304	0.9746
Sentence-BERT	0.152	0.9315	0.9301	0.9308	0.9724

2 HUNGARIAN SAMPLES

Table S5. Hungarian synonyms for smoking.

bagarettázik	cigarettacsutka	dohányzik	pipás	szipákol
bagó	cigarettavég	dohányzás	pipázik	szivar
bagós	cigarettázik	eregeti a füstöt	pipázó	szivarka
bagózik	cigarettázó	füstöl	pöfékel	szivarozik
bagózó	cigi	füstölnivaló	rigó	szivarozó
bláz	cigizik	királydekk	rágyújt	szí
bűzrúd	csikk	koporsószeg	spanglizik	szív
cigaretta	dohány	mint a gyárkémény	staub	
cigarettacsikk	dohányos	pipál	staubol	

Table S6. A Hungarian example prompt for ChatGPT.

Generálj dohányzással kapcsolatos szöveget.
A szöveg különböző mondatokban mindenképpen taralmazza az alábbi szavakat:
dohányzás, pipál, dohány, szivar, cigaretta.

Table S7. Paragraph generated by the Table S6 prompt.

A dohányzás és a szivarozás olyan szokások,
amelyeket sok ember szeret gyakorolni.
A Karelia, a szivar és a pipa a legnépszerűbb dohánytermékek a piacon.
A dohánytermékek általában drágábbak, mint a hagyományos cigaretták,
de sok ember szerint jobb minőségűek is.
A szivarozás egy másik lehetőség,
amelyet sok ember választ, mivel kevesebb egészségügyi kockázattal jár,
mint a hagyományos cigaretták.
Azonban a szivarozás használata nem teljesen biztonságos,
és további kutatásokra van szükség annak megállapításához,
hogy milyen hatással van az egészségre.
Azonban a szivarozás használata sokkal kevésbé káros,
mint a hagyományos cigaretták.

Table S8. Generated paragraph example.

A **dohányzás** sajnos számos káros anyagot tartalmaz, amelyek károsítják az egészséget. A **csikk, szivarozó** és **cigi** sem segít ezen. Ha szeretnél egészségesebb életmódot folytatni, érdemes leszokni a **dohányzásról.** Azonban tudjuk, hogy ez nem mindig egyszerű. Ha szeretnél segítséget, kérj tanácsot orvosodtól vagy keresd fel a **dohányzás** leszokást segítő szervezeteket.

Table S9. Generated paragraph example.

Sokan nem tudják abbahagyni a bűzrudat, pedig tudják, hogy milyen káros hatása van. Nemcsak maguknak ártanak vele, hanem másoknak is. Aki mint a gyárkémény **füstöl** nap mint nap, az nemcsak saját tüdejét roncsolja le, hanem másokét is. A **passzív dohányosok** ugyanolyan veszélynek vannak kitéve, mint az aktív **dohányosok.** Nem mindegy tehát, hogy hol és mikor gyújt rá valaki. Aki **pipázik** vagy **cigarettázik** mellettünk egy zárt térben vagy egy tömegközlekedési eszközön, az nemcsak udvariatlan és tiszteletlen velünk szemben, hanem egészségünket is veszélyezteti. A legjobb dolog tehát az lenne, ha mindenki leszokna erről a rossz szokásról. De ha ez nem megy könnyen, akkor legalább próbáljon meg odafigyelni arra, hogy ne zavarja vele másokat. És persze ne feledje: soha nem késő elkezdeni a leszokást!

Table S10. Generated paragraph example.

Minden reggel ugyanazt csinálja: felkel, **rágyújt** egy **cigire** és elindul dolgozni. Nem törődik vele, hogy milyen kárt okoz magának ezzel. Pedig tudnia kellene, hogy minden egyes koporsószeg rövidíti az életét. Nemcsak őt veszélyezteti ezzel a rossz szokással, hanem családját és barátait is. Aki mellette van, az is belélegzi a mérgező füstöt. Aki szereti őt, az aggódik érte. De ő nem hallgat senkire. Úgy gondolja, hogy ez az ő döntése és senki sem mondhatja meg neki mit tegyen vagy ne tegyen. Pedig nem lenne nehéz leszoknia erről a **bláz** dologról. Sokféle módszer létezik erre: gyógyszerek, tapaszok, rágógumik vagy akár hipnózis is segíthet abban, hogy megszabaduljon ettől a szenvedélytől. De ha ezek nem hatnak rá eléggé akkor ott vannak még más lehetőségek is: például az elektromos **cigaretta** vagy a **dohánymentes** töltetek használata. Ezekkel legalább csökkenthetné az egészségkockázatot és javíthatná az életminőségét. Nem kellene mindig **bagós** legyen és büdös lennie.

Table S11. Generated paragraph example.

A **dohányzás** során a **dohányosok rágyújtanak** és **dohányoznak,** miközben eregetik a füstöt. A **cigarettacsutka** is káros hatású. A **dohányzás** során a **dohányosok** belélegzik a **dohányt,** amely káros hatással van az egészségre. A **dohányzásról** való leszokás az egészség javítása érdekében fontos. A **dohányzásról** való leszokás nehéz lehet, de az egészség javítása érdekében érdemes megpróbálni. A **dohányzásról** való leszokás előnyei közé tartozik a jobb egészség, a jobb fizikai állapot és a jobb életminőség.

Table S12. Generated paragraph example.

A **dohányzás** káros az egészségre, és a **cigizőknek** érdemes lenne leszokniuk. A **szivarka** és a pipa is káros, de a bagarettázók is érdemes lenne leszokniuk.

Table S13. Generated paragraph example.

A bagarettázás, a **rigó,** a **pipás** és a **szivar** mind olyan szavak, amelyek a **dohányzással** kapcsolatosak. A spanglizás pedig egy olyan tevékenység, amelyet a **dohányosok** gyakran űznek. Azonban a **dohányzás** káros hatásai miatt mindenkinek érdemes megfontolnia a leszokást. A **dohányzás** ugyanis nem csak a **dohányosok** egészségére, hanem a környezetre is káros hatással van.

Table S14. Generated paragraph example.

A szív az egyik legfontosabb szervünk, amelyet a **dohányzás** károsíthat. A **királydekk,** a **szipákolás,** a **cigarettacsutka** és a mint a gyárkémény mind olyan szavak, amelyek a **dohányzással** kapcsolatosak. Azonban a **dohányzás** káros hatásai miatt mindenkinek érdemes megfontolnia a leszokást.

Table S15. Generated paragraph example.

A **dohányzás** olyan, mint a gyárkémény, amely füstöt bocsát ki. A **pöfékelés** és a **blázolás** is káros hatású. A **dohányzás** során a **dohányosok** belélegzik a **dohányt,** amely káros hatással van az egészségre. A **cigaretta** is káros hatású. A **dohányzásról** való leszokás az egészség javítása érdekében fontos. A **dohányzásról** való leszokás nehéz lehet, de az egészség javítása érdekében érdemes megpróbálni. A **dohányzásról** való leszokás előnyei közé tartozik a jobb egészség, a jobb fizikai állapot és a jobb életminőség.

Table S16. Generated paragraph example.

A **szivar** és a **királydekk** is káros hatású. A **dohányzás** során a **dohányosok** belélegzik a **csikket,** amely káros hatással van az egészségre. A bagarettázás is káros hatású. A **dohányzás** során a **dohányosok** belélegzik a **dohányt,** amely káros hatással van az egészségre. A **dohányzásról** való leszokás az egészség javítása érdekében fontos. A **dohányzásról** való leszokás nehéz lehet, de az egészség javítása érdekében érdemes megpróbálni.

3 TEST DATA IN HUNGARIAN FOR THE NAMED ENTITY RECOGNITION

Büdös. Káros. Ultraciki. Ne **szívd** (meg)! A folyamatosan tartó, enyhén mérgezett állapot A **dohányzás** nemcsak egy rossz szokás, de előrehaladottabb stádiumában krónikus betegség is. Mint minden függőségnél, ennél is fellép a sóvárgás érzése, ami csak azután csillapodik, hogy a szervezet újabb nikotinadaghoz jut. Szinte mindenkinek van valamilyen véleménye a **dohányzásról**. Noha elfogadottsága egyre csökken, a KSH 2019-es adatai szerint Magyar-országon a felnőtt lakosságnak még így is a 27 százaléka **dohányzik**. Ez pedig a **dohányzó** személy mellett hatással van mindazok egészségi állapotára is, akik a környezetében elszenvedik ennek a káros szokásnak a következményeit. A legveszélyesebb 18 éves kor előtt rászokni, hiszen ekkor alakul ki a leg-súlyosabb függőség. Mi van a **cigiben**? A **cigarettában** és annak az égés során keletkező **füstjében** körülbelül 4000 különböző anyag található, ebből 70 bizonyítottan rákkeltő (csak néhány azon anyagok közül, amelyek az egészséget károsítják: benzol, kadmium, ammónia, hidrogén-cianid, aceton, DDT, formaldehid, polónium). A nikotin (vagy az alternatív termékekben: nikotin só) minden **dohánytermékben** jelen van. Ez az erős vegyület egy legális drog, amely a függőség kialakulásáért és fenntartásáért felel. Halálos dózisa 20–60 mg (1 szál cigaretta átlagosan 1 mg nikotint tartalmaz). Számos módon hat a szervezetre: aktiválja a szimpatikus idegrendszert, szűkíti az artériákat, növeli a vérnyomást és a pulzust, emeli a vércukor- és koleszterinszintet. Annyira káros, hogy rovarirtóként is kiválóan alkalmazható. A vér nikotinszintje kb. 1,5–2 óra alatt feleződik, általában ennyi idővel az utolsó cigaretta elszívása után jelentkezik a sóvárgás. Ha a szervezet ilyenkor nem jut hozzá, akkor a **dohányos** ingerlékenyebb, nyugtalanabb és dekoncentráltabb lehet, esetleg gyakrabban érzi magát éhesnek. Ezek az első megvonási tünetek, amelyek szerencsére leszokást követően pár nap, esetleg néhány hét alatt enyhülnek. A **dohánytermékekben** található anyagok közül, sokaknak eszébe jut a nikotin mellett a kátrány, valamint a szén-monoxid is. Az előbbi égése okozza a jellegzetes, sokak számára büdös szagot, ez rakódik le a légzőrendszerben, valamint ez az, ami csökkenti a tüdő aktív légzőfelületét is. A szén-monoxid hatása sem kedvezőbb, hiszen ez a mérgező gáz akadályozza a szervezet oxigénellátását. Hatása rövidtávon is érzékelhető: például az első **cigaretta** elszívása során fellépő fejfájás, émelygés, hányinger és szédülés is a számlájára írható. Ami az alternatív **dohány-termékeket** illeti: ezek ugyanúgy, egytől egyig megrövidítik az életet! Fenntartják a nikotinfüggőséget és közvetlenül toxikus anyagokat adagolnak a szervezetbe. Senkit ne tévesszen meg az, hogy dizájnosabbak a készülékek, illetve trendibb a csomagolás! Aki szívja, az előbb utóbb megszívja! A passzív dohányzás. A káros hatásoknak nemcsak a **dohányos** van kitéve, hanem annak környezete – köztük a **nemdohányzók** – is. Az úgynevezett **főfüstöt** – az ebben található összes káros anyaggal és égéstermékkel – a **dohányos** direkt módon szívja be a tüdejébe. Emellett az égő **cigarettából**, **vízipipából**, **elektromos cigarettából** közvetlenül a levegőbe távozik a mellékfüst, amelyben – mivel ezt filter sem szűri – még több káros égéstermékek van. A kifújt **füstöt** és a **mellékfüstöt** nemcsak a **dohányzó szívja** be, hanem a közelében lévők is. Ezt nevezik passzív **dohányzásnak**, elszenvedői pedig legtöbbször a családtagok, a munkatársak és a házi kedvencek. Van egy kevésbé ismert hatás is, ez a harmadlagos **dohányzás**. Kimutatható, hogy a füstben lévő toxikus anyagok káros hatásukat a tárgyakra ülepedve még sok-sok éven keresztül kifejtik. A nikotinmentes élet jobb és egészségesebb élet. A legjobb az, ha soha rá sem szokunk. A második legjobb dolog pedig az, ha valaki most leszokik. Mindkettő döntő mértékben befolyásolhatja az életminőséget és a további egészségben eltölthető életévek számát. Csökken a szívinfarktus, a stroke, szájüregi daganatok, a bőr- és a tüdőrák kialakulásának kockázata. A **dohányzás** elhagyása szebbé teszi a bőrt és egészségesebbé a fogakat, továbbá a test illatára is pozitívan hat. A **nemdohányzó** embernek több az energiája, a mindennapok során nagyobb teljesítményre képes – így teljesebb életet élhetünk. Le lehet tenni! Mivel a **dohányzás** egy tanult viselkedés, a leszokást nemcsak a nikotinfüggőség, hanem a kialakult szokásrendszer is megnehezíti. A sikeres elhagyáshoz fel kell tárni a **dohányzáshoz** kapcsolódó érzelmeket, gondolatokat és motivációkat. A leszokás időigényes feladat, mert

az új életmód erős elkötelezettség mellett is 3–6 hónap alatt válik napi rutinná. A tiszta állapot fenntartása nagy önfegyelmet igényel. Noha a **dohányzás** elhagyása után a kapcsolódó nikotinfüggő receptorok aktivitása csökken, később akár egyetlen szál **cigaretta** is újra aktívvá teszi őket, vagyis egy megingás elég lehet a visszaeséshez. A leszokáshoz szakszerű támogatás kérhető a háziorvostól, illetve, ha szükséges, pszichológustól. Érdemes mielőbb belevágni! Jobb, ha a leszokást az egészségesebb élet vágya vezérli és nem csupán a már romló, sokszor nem helyreállítható egészségi állapot!

4 MULTIMODAL FILTERING ALGORITHM

Algorithm 1 Multimodal filtering

$S \leftarrow clip.encode(\text{"}smoking\text{"})$
$I \leftarrow preprocess(video)$ ▷ Processing of videos and images
$IE \leftarrow clip.encode(I)$ ▷ Encoding images
$CS \leftarrow cosine.similarity(S, IE)$ ▷ Calculation of cosine similarity
$c = 0$ ▷ Correction constant
$cl \leftarrow mean(CS) + c$ ▷ Calculation of the cutting line
$CS \leftarrow order(CS)$ ▷ Sorting in ascending order
$n \leftarrow len(CS)$
while $n \neq 0$ **do**
  if $CS[n] \geq cl$ **then**
    $I \leftarrow drop(I, n)$ ▷ Dropping image from I array
  end if
  $n \leftarrow n - 1$
end while
